# Tetra­kis(2-amino­pyrazine-κ*N* ^4^)dichlorido­cobalt(II)

**DOI:** 10.1107/S1600536809045309

**Published:** 2009-11-04

**Authors:** Wei Kang, Li-Hua Huo, Shan Gao, Seik Weng Ng

**Affiliations:** a College of Chemistry and Materials Science, Heilongjiang University, Harbin 150080, People’s Republic of China; b Department of Chemistry, University of Malaya, 50603 Kuala Lumpur, Malaysia

## Abstract

The Co^II^ atom in the title complex, [CoCl_2_(C_4_H_5_N_3_)_4_], exists in an all-*trans* Cl_2_N_4_Co octa­hedral geometry. The Co^II^ atom lies on a special position of 2 site symmetry. Adjacent mol­ecules are linked by N—H⋯N and N—H⋯Cl hydrogen bonds into a three-dimensional network.

## Related literature

For the triclinic modification, see: Csöregh *et al.* (2000 ▶).
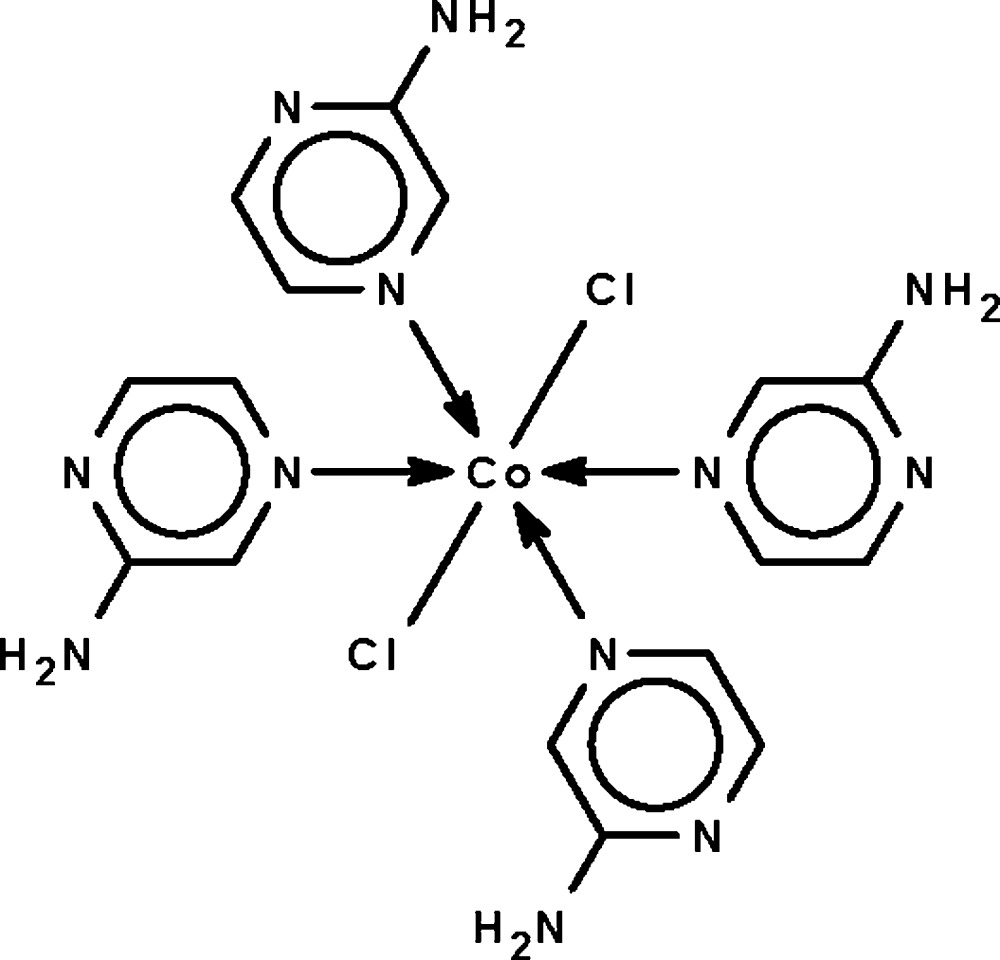


## Experimental

### 

#### Crystal data

[CoCl_2_(C_4_H_5_N_3_)_4_]
*M* *_r_* = 510.27
Orthorhombic, 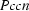

*a* = 7.6347 (2) Å
*b* = 15.7341 (4) Å
*c* = 18.6074 (4) Å
*V* = 2235.22 (9) Å^3^
*Z* = 4
Mo *K*α radiation
μ = 1.04 mm^−1^
*T* = 293 K
0.30 × 0.20 × 0.15 mm

#### Data collection

Rigaku R-AXIS RAPID IP diffractometer
Absorption correction: multi-scan (*ABSCOR*; Higashi, 1995 ▶) *T*_min_ = 0.746, *T*_max_ = 0.860
20053 measured reflections
2553 independent reflections
2234 reflections with *I* > 2σ(*I*)
*R*_int_ = 0.024

#### Refinement

*R*[*F* ^2^ > 2σ(*F* ^2^)] = 0.025
*wR*(*F* ^2^) = 0.074
*S* = 1.06
2553 reflections
157 parameters
4 restraints
H atoms treated by a mixture of independent and constrained refinement
Δρ_max_ = 0.29 e Å^−3^
Δρ_min_ = −0.25 e Å^−3^

### 

Data collection: *RAPID-AUTO* (Rigaku, 1998 ▶); cell refinement: *RAPID-AUTO*; data reduction: *CrystalClear* (Rigaku/MSC, 2002 ▶); program(s) used to solve structure: *SHELXS97* (Sheldrick, 2008 ▶); program(s) used to refine structure: *SHELXL97* (Sheldrick, 2008 ▶); molecular graphics: *X-SEED* (Barbour, 2001 ▶); software used to prepare material for publication: *publCIF* (Westrip, 2009 ▶).

## Supplementary Material

Crystal structure: contains datablocks global, I. DOI: 10.1107/S1600536809045309/xu2656sup1.cif

Structure factors: contains datablocks I. DOI: 10.1107/S1600536809045309/xu2656Isup2.hkl

Additional supplementary materials:  crystallographic information; 3D view; checkCIF report

## Figures and Tables

**Table 1:** Selected bond lengths (Å)

Co1—N1	2.2068 (11)
Co1—N4	2.1941 (11)
Co1—Cl1	2.4206 (4)

**Table 2:** Hydrogen-bond geometry (Å, °)

*D*—H⋯*A*	*D*—H	H⋯*A*	*D*⋯*A*	*D*—H⋯*A*
N3—H1⋯Cl1^i^	0.85 (1)	2.36 (1)	3.209 (2)	175 (2)
N3—H2⋯N5^ii^	0.86 (1)	2.43 (2)	3.134 (2)	140 (2)
N6—H3⋯Cl1^iii^	0.85 (1)	2.42 (1)	3.265 (1)	171 (2)
N6—H4⋯N2^iv^	0.86 (1)	2.33 (2)	3.045 (2)	142 (2)

Symmetry codes: (i) 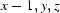; (ii) 
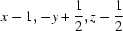
; (iii) 
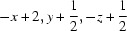
; (iv) 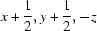.

**Fig. 1.**
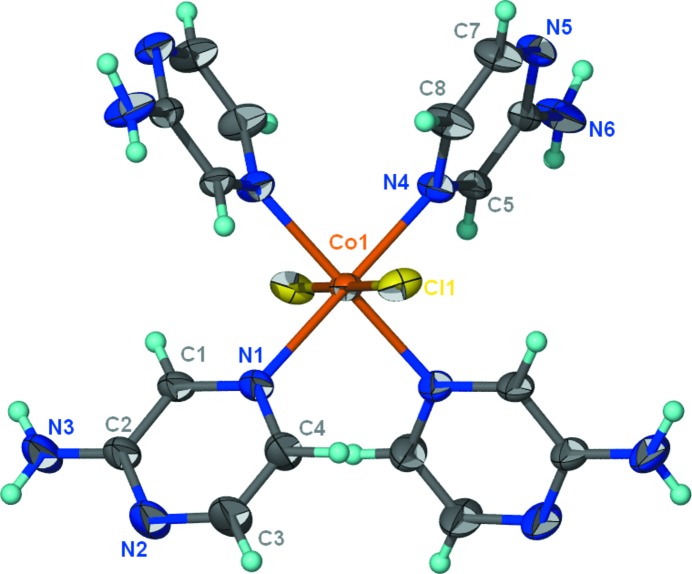
Thermal ellipsoid plot (Barbour, 2001) of CoCl2(C2H5N3)4 at the 50% probability level; hydrogen atoms are drawn as spheres of arbitrary radius.

## supplementary crystallographic information

### Experimental

Cobalt(II) chloride hexahydrate (0.48 g, 2 mmol) and 2-aminopyrazine (0.19 g, 2 mmol) were dissolved in a small volume of water. Red crystals of the adduct separated from the filtered solution after several days. CH&N elemental analysis. Calc. for C_16_H_20_Cl_2_N_12_Co: C 37.66, H 3.95, N 32.94%; found: C 37.63, H 3.89, N 32.97%.

### Refinement

Amino-H atoms were located in a difference Fourier map and refined isotropically with a distance restraint of N–H = 0.86±0.01 Å. Carbon-bound H-atoms were placed in calculated positions (C—H = 0.93 Å) and were included in the refinement in the riding model approximation with U_iso_(H) set to 1.2U_eq_(C).

### Crystal data

**Table:**

[CoCl_2_(C_4_H_5_N_3_)_4_]	*F*(000) = 1044
*M**_r_* = 510.27	*D*_x_ = 1.516 Mg m^−^^3^
Orthorhombic, *P**c**c**n*	Mo *K*α radiation, λ = 0.71073 Å
Hall symbol: -P 2ab 2ac	Cell parameters from 15790 reflections
*a* = 7.6347 (2) Å	θ = 2.3–27.5°
*b* = 15.7341 (4) Å	µ = 1.04 mm^−^^1^
*c* = 18.6074 (4) Å	*T* = 293 K
*V* = 2235.22 (9) Å^3^	Block, red
*Z* = 4	0.30 × 0.20 × 0.15 mm

### Data collection

**Table:**

Rigaku RAXIS-RAPID IP diffractometer	2553 independent reflections
Radiation source: fine-focus sealed tube	2234 reflections with *I* > 2σ(*I*)
graphite	*R*_int_ = 0.024
ω scans	θ_max_ = 27.5°, θ_min_ = 3.2°
Absorption correction: multi-scan (*ABSCOR*; Higashi, 1995)	*h* = −9→9
*T*_min_ = 0.746, *T*_max_ = 0.860	*k* = −20→20
20053 measured reflections	*l* = −24→24

### Refinement

**Table:**

Refinement on *F*^2^	Primary atom site location: structure-invariant direct methods
Least-squares matrix: full	Secondary atom site location: difference Fourier map
*R*[*F*^2^ > 2σ(*F*^2^)] = 0.025	Hydrogen site location: inferred from neighbouring sites
*wR*(*F*^2^) = 0.074	H atoms treated by a mixture of independent and constrained refinement
*S* = 1.06	*w* = 1/[σ^2^(*F*_o_^2^) + (0.0439*P*)^2^ + 0.4514*P*] where *P* = (*F*_o_^2^ + 2*F*_c_^2^)/3
2553 reflections	(Δ/σ)_max_ = 0.001
157 parameters	Δρ_max_ = 0.28 e Å^−^^3^
4 restraints	Δρ_min_ = −0.25 e Å^−^^3^

### Fractional atomic coordinates and isotropic or equivalent isotropic displacement parameters (Å^2^)

**Table:**

	*x*	*y*	*z*	*U*_iso_*/*U*_eq_	
Co1	0.7500	0.2500	0.103443 (12)	0.02794 (9)	
Cl1	0.98632 (5)	0.14744 (2)	0.103952 (17)	0.03970 (11)	
N1	0.61989 (16)	0.17473 (8)	0.01880 (6)	0.0348 (3)	
N2	0.46194 (19)	0.08976 (11)	−0.09614 (7)	0.0553 (4)	
N3	0.1968 (2)	0.09877 (14)	−0.03954 (8)	0.0686 (5)	
N4	0.88907 (16)	0.31841 (7)	0.18890 (6)	0.0339 (3)	
N5	1.03169 (17)	0.40550 (8)	0.30631 (6)	0.0387 (3)	
N6	0.9535 (2)	0.53209 (8)	0.25231 (7)	0.0527 (4)	
C1	0.45050 (19)	0.15892 (9)	0.01846 (7)	0.0357 (3)	
H1A	0.3823	0.1771	0.0569	0.043*	
C2	0.3693 (2)	0.11475 (10)	−0.03920 (7)	0.0413 (3)	
C3	0.6338 (2)	0.10573 (14)	−0.09351 (9)	0.0625 (6)	
H3A	0.7027	0.0878	−0.1318	0.075*	
C4	0.7141 (2)	0.14683 (13)	−0.03790 (9)	0.0509 (4)	
H4A	0.8345	0.1557	−0.0391	0.061*	
C5	0.88843 (19)	0.40188 (8)	0.19189 (6)	0.0319 (3)	
H5	0.8395	0.4324	0.1541	0.038*	
C6	0.96002 (18)	0.44673 (8)	0.25094 (7)	0.0325 (3)	
C7	1.0327 (2)	0.32043 (10)	0.30155 (8)	0.0453 (4)	
H7	1.0823	0.2896	0.3390	0.054*	
C8	0.9645 (2)	0.27664 (10)	0.24459 (8)	0.0452 (4)	
H8	0.9700	0.2176	0.2440	0.054*	
H1	0.136 (3)	0.1094 (14)	−0.0022 (8)	0.078 (7)*	
H2	0.155 (3)	0.0704 (11)	−0.0753 (8)	0.063 (6)*	
H3	0.978 (3)	0.5580 (12)	0.2913 (7)	0.060 (6)*	
H4	0.909 (2)	0.5583 (11)	0.2164 (8)	0.061 (6)*	

### Atomic displacement parameters (Å^2^)

**Table:**

	*U*^11^	*U*^22^	*U*^33^	*U*^12^	*U*^13^	*U*^23^
Co1	0.03367 (16)	0.02901 (14)	0.02114 (14)	−0.00229 (9)	0.000	0.000
Cl1	0.0462 (2)	0.0432 (2)	0.02968 (18)	0.01013 (15)	0.00662 (13)	0.00476 (12)
N1	0.0337 (6)	0.0435 (6)	0.0271 (5)	−0.0030 (5)	0.0015 (5)	−0.0079 (5)
N2	0.0407 (8)	0.0851 (11)	0.0401 (7)	−0.0055 (7)	0.0052 (6)	−0.0322 (7)
N3	0.0366 (8)	0.1287 (16)	0.0407 (8)	−0.0134 (9)	0.0027 (6)	−0.0368 (9)
N4	0.0428 (7)	0.0326 (5)	0.0262 (5)	−0.0011 (5)	−0.0058 (5)	0.0003 (4)
N5	0.0447 (7)	0.0433 (6)	0.0281 (5)	−0.0005 (5)	−0.0095 (5)	−0.0004 (5)
N6	0.0912 (12)	0.0339 (6)	0.0331 (6)	−0.0092 (7)	−0.0211 (7)	0.0002 (5)
C1	0.0359 (7)	0.0464 (7)	0.0248 (6)	0.0013 (6)	0.0028 (5)	−0.0084 (6)
C2	0.0352 (8)	0.0588 (9)	0.0300 (6)	−0.0012 (7)	0.0005 (6)	−0.0124 (6)
C3	0.0416 (9)	0.1031 (16)	0.0429 (9)	−0.0042 (10)	0.0106 (7)	−0.0368 (9)
C4	0.0340 (8)	0.0789 (12)	0.0398 (8)	−0.0060 (7)	0.0075 (6)	−0.0206 (8)
C5	0.0399 (7)	0.0323 (6)	0.0236 (6)	−0.0039 (5)	−0.0055 (5)	0.0036 (5)
C6	0.0376 (7)	0.0349 (6)	0.0251 (6)	−0.0057 (5)	−0.0034 (5)	0.0008 (5)
C7	0.0582 (10)	0.0450 (8)	0.0326 (7)	0.0103 (7)	−0.0151 (7)	0.0028 (6)
C8	0.0650 (11)	0.0335 (7)	0.0371 (7)	0.0090 (7)	−0.0136 (7)	0.0015 (6)

### Geometric parameters (Å, °)

**Table:**

Co1—N1^i^	2.2068 (11)	N5—C6	1.3348 (18)
Co1—N1	2.2068 (11)	N5—C7	1.341 (2)
Co1—N4	2.1941 (11)	N6—C6	1.3441 (18)
Co1—N4^i^	2.1941 (11)	N6—H3	0.852 (9)
Co1—Cl1^i^	2.4206 (4)	N6—H4	0.856 (9)
Co1—Cl1	2.4206 (4)	C1—C2	1.4207 (19)
N1—C1	1.3170 (19)	C1—H1A	0.9300
N1—C4	1.3501 (19)	C3—C4	1.366 (2)
N2—C2	1.3332 (19)	C3—H3A	0.9300
N2—C3	1.337 (2)	C4—H4A	0.9300
N3—C2	1.341 (2)	C5—C6	1.4157 (18)
N3—H1	0.853 (9)	C5—H5	0.9300
N3—H2	0.864 (9)	C7—C8	1.367 (2)
N4—C5	1.3145 (17)	C7—H7	0.9300
N4—C8	1.3556 (18)	C8—H8	0.9300
			
N4—Co1—N4^i^	87.12 (6)	C6—N6—H4	118.8 (14)
N4—Co1—N1^i^	92.07 (4)	H3—N6—H4	121 (2)
N4^i^—Co1—N1^i^	176.67 (4)	N1—C1—C2	121.63 (13)
N4—Co1—N1	176.67 (4)	N1—C1—H1A	119.2
N4^i^—Co1—N1	92.07 (4)	C2—C1—H1A	119.2
N1^i^—Co1—N1	88.93 (6)	N2—C2—N3	117.51 (14)
N4—Co1—Cl1^i^	91.77 (3)	N2—C2—C1	120.88 (15)
N4^i^—Co1—Cl1^i^	87.91 (3)	N3—C2—C1	121.59 (13)
N1^i^—Co1—Cl1^i^	88.89 (3)	N2—C3—C4	123.88 (15)
N1—Co1—Cl1^i^	91.43 (3)	N2—C3—H3A	118.1
N4—Co1—Cl1	87.91 (3)	C4—C3—H3A	118.1
N4^i^—Co1—Cl1	91.77 (3)	N1—C4—C3	120.47 (15)
N1^i^—Co1—Cl1	91.43 (3)	N1—C4—H4A	119.8
N1—Co1—Cl1	88.89 (3)	C3—C4—H4A	119.8
Cl1^i^—Co1—Cl1	179.552 (18)	N4—C5—C6	121.97 (12)
C1—N1—C4	117.25 (12)	N4—C5—H5	119.0
C1—N1—Co1	123.15 (9)	C6—C5—H5	119.0
C4—N1—Co1	119.50 (10)	N5—C6—N6	119.10 (12)
C2—N2—C3	115.85 (13)	N5—C6—C5	121.00 (12)
C2—N3—H1	119.6 (16)	N6—C6—C5	119.89 (12)
C2—N3—H2	117.9 (14)	N5—C7—C8	123.50 (13)
H1—N3—H2	122 (2)	N5—C7—H7	118.2
C5—N4—C8	116.97 (12)	C8—C7—H7	118.2
C5—N4—Co1	121.27 (9)	N4—C8—C7	120.67 (14)
C8—N4—Co1	121.45 (10)	N4—C8—H8	119.7
C6—N5—C7	115.87 (12)	C7—C8—H8	119.7
C6—N6—H3	119.0 (14)		
			
N4^i^—Co1—N1—C1	45.73 (12)	C3—N2—C2—N3	−179.1 (2)
N1^i^—Co1—N1—C1	−131.09 (13)	C3—N2—C2—C1	2.7 (3)
Cl1^i^—Co1—N1—C1	−42.23 (11)	N1—C1—C2—N2	−1.9 (2)
Cl1—Co1—N1—C1	137.45 (11)	N1—C1—C2—N3	−179.93 (17)
N4^i^—Co1—N1—C4	−138.03 (13)	C2—N2—C3—C4	−1.6 (3)
N1^i^—Co1—N1—C4	45.15 (12)	C1—N1—C4—C3	1.4 (3)
Cl1^i^—Co1—N1—C4	134.01 (13)	Co1—N1—C4—C3	−175.03 (16)
Cl1—Co1—N1—C4	−46.30 (13)	N2—C3—C4—N1	−0.5 (4)
N4^i^—Co1—N4—C5	−124.52 (13)	C8—N4—C5—C6	−1.1 (2)
N1^i^—Co1—N4—C5	52.25 (11)	Co1—N4—C5—C6	172.58 (10)
Cl1^i^—Co1—N4—C5	−36.70 (11)	C7—N5—C6—N6	179.54 (15)
Cl1—Co1—N4—C5	143.61 (11)	C7—N5—C6—C5	0.7 (2)
N4^i^—Co1—N4—C8	48.91 (11)	N4—C5—C6—N5	0.1 (2)
N1^i^—Co1—N4—C8	−134.32 (13)	N4—C5—C6—N6	−178.74 (15)
Cl1^i^—Co1—N4—C8	136.73 (12)	C6—N5—C7—C8	−0.5 (3)
Cl1—Co1—N4—C8	−42.96 (12)	C5—N4—C8—C7	1.4 (2)
C4—N1—C1—C2	−0.3 (2)	Co1—N4—C8—C7	−172.29 (13)
Co1—N1—C1—C2	176.01 (11)	N5—C7—C8—N4	−0.6 (3)

Symmetry codes: (i) −*x*+3/2, −*y*+1/2, *z*.

### Hydrogen-bond geometry (Å, °)

**Table:**

*D*—H···*A*	*D*—H	H···*A*	*D*···*A*	*D*—H···*A*
N3—H1···Cl1^ii^	0.85 (1)	2.36 (1)	3.209 (2)	175 (2)
N3—H2···N5^iii^	0.86 (1)	2.43 (2)	3.134 (2)	140 (2)
N6—H3···Cl1^iv^	0.85 (1)	2.42 (1)	3.265 (1)	171 (2)
N6—H4···N2^v^	0.86 (1)	2.33 (2)	3.045 (2)	142 (2)

Symmetry codes: (ii) *x*−1, *y*, *z*; (iii) *x*−1, −*y*+1/2, *z*−1/2; (iv) −*x*+2, *y*+1/2, −*z*+1/2; (v) *x*+1/2, *y*+1/2, −*z*.
